# The potential benefit of scaling up malaria prevention to reduce low birth weight in Africa

**DOI:** 10.1371/journal.pmed.1002244

**Published:** 2017-02-28

**Authors:** James G. Beeson, Julie A. Simpson

**Affiliations:** 1 Burnet Institute, Melbourne, Victoria, Australia; 2 Monash University, Central Clinical School and Department of Microbiology, Monash University, Australia; 3 Centre for Epidemiology and Biostatistics, Melbourne School of Population and Global Health and Department of Medicine, The University of Melbourne, Melbourne, Australia

## Introduction

Low birth weight (LBW) is one of the most important risk factors for neonatal mortality and morbidity, childhood stunting, reduced cognitive development, and chronic diseases later in life. In sub-Saharan Africa, where the burden of LBW is very high, *Plasmodium falciparum* malaria is one of the major causes, contributing to an estimated 20% of LBW cases [1]. A new report suggests that substantial gains in reducing LBW in sub-Saharan Africa could be achieved by scaling up a key malaria intervention known as intermittent preventive treatment in pregnancy (IPTp) [2].

In areas with stable *P*. *falciparum* malaria transmission, IPTp is a key strategy for reducing the burden of malaria in pregnancy and subsequent LBW; other important strategies are widespread deployment of long-lasting insecticide-treated bed nets (ITNs) and access to prompt diagnosis and effective treatment. IPTp involves the administration of curative doses of sulphadoxine-pyrimethamine (SP) to pregnant women attending antenatal clinics (ANC) without testing for malaria infection. IPTp has shown substantial benefit in reducing LBW and severe maternal anaemia (pooled risk ratio from multiple trials was 0.8 and 0.6, respectively) [3]. Its benefits are believed to stem from the clearance of any infection that may be present when the drug is given and a short-lived prophylactic effect against subsequent *P*. *falciparum* infection because of the drugs’ long half-lives (4–9 days for sulphadoxine and ~3 days for pyrimethamine [4].

Since 2004, WHO has recommended IPTp for all pregnant women in areas of stable malaria transmission in addition to the use of ITNs [5]. The initial recommendation was at least two separate doses, and monthly doses in pregnancy are now recommended where possible. Despite this, IPTp coverage in at-risk populations in sub-Saharan Africa is very low in many regions; overall, an estimated 24% of women currently receive at least two doses [6]. Furthermore, coverage with ITNs during pregnancy is similarly low at an estimated 35% in at-risk populations [2]. Of additional concern is the declining efficacy of IPTp with SP due to increasing resistance in many regions in Africa (particularly resistance caused by six specific mutations) [7].

## Modelling the potential benefit of enhanced IPTp coverage

In new research published in *PLOS Medicine*, Walker and colleagues performed a comprehensive mathematical modelling study to provide estimates of LBW deliveries that would be averted if IPTp SP coverage were to be substantially increased in sub-Saharan Africa [2]. In their analysis, they allowed for areas with limited SP effectiveness due to drug resistance and declining malaria transmission and also considered the effect of ITN coverage. Their findings indicate that enormous health gains can be achieved through maximizing IPTp coverage despite concerns regarding SP resistance or declining malaria burden in some regions. Their modelling estimated an additional 215,000 LBW deliveries could be prevented each year if IPTp SP were to be administered to all women attending at least three ANC visits in malaria-endemic regions of sub-Saharan Africa. Their modelling also found that IPTp scale-up would have major benefits even with good ITN coverage. Others have reported the benefits of IPT in children in the context of good ITN coverage [8], supporting the concept that there is benefit in implementing both interventions.

Walker and colleagues relied on four data sources [2]. They extracted data on IPTp SP coverage and ANC attendance from the most recent Demographic Health Surveys or Malaria Indicator Surveys (2010–2014). Estimates of malaria prevalence (years 2000–2015) were obtained from the Malaria Atlas Project, and the prevalence estimates of SP resistance (K540E [quintuple mutation] and A581G [sextuple mutation], year 2010) were obtained from the Worldwide Antimalarial Resistance Network molecular database. The efficacy of IPTp SP was calculated from a number of trials conducted prior to adoption of IPTp SP policy, and the impact of resistance on SP efficacy was included explicitly in their model. As with all mathematical modelling studies, there are always questions surrounding whether the model captures all the salient factors and the accuracy of the data sources used to provide the model inputs. Due to computational issues, the sensitivity of the projected estimate of averted LBW deliveries to uncertainty in the estimated values was only explored for the estimates of IPTp SP efficacy. Therefore, it is possible that the projected figure of 215,000 averted LBW deliveries could be an under- or overestimation. Furthermore, as noted by the authors, updated figures on the prevalence of SP resistance and IPTp SP efficacy are desirable to improve estimates.

It should also be noted that the study modelled malaria in Africa, where the major burden of malaria occurs, caused predominantly by *P*. *falciparum*, and did not include other regions. There is a large burden of malaria in pregnancy in Asia, the Pacific, and the Americas, where *Plasmodium vivax* causes a large proportion of malaria; in many regions in Asia, *P*. *falciparum* resistance rates for SP are much higher. Prevention of malaria in pregnancy in these regions requires different drugs or alternate strategies.

## Concerns regarding IPTp in the face of increasing drug resistance

Calls for the scale-up of IPTp SP coverage have not been without criticisms [9]. A key concern relates to declining efficacy of SP as a result of increasing drug resistance. Some data suggested that SP use in populations with high levels of resistance is associated with worse pregnancy outcomes [7], but this has not been a consistent finding [10]. The modelling by Walker et al. considered SP resistance and found that there was still a major benefit of IPTp in Africa because the major burden occurs in regions where SP efficacy remains high. They estimated that SP would have greatly reduced efficacy in 25% of the at-risk population where high-level resistance occurs.

Clearly though, in the face of increasing resistance and the large population affected by drug-resistant malaria, replacement drugs for IPTp and additional strategies for prevention need to be established and implemented. Alternative drugs for IPTp include dihydroartemisinin-piperaquine, which was superior to SP in recent clinical trials in regions with significant SP resistance [11] [12]. An alternative approach of intermittent screening of women for malaria at ANC followed by treatment of positive cases has also been investigated; however, results suggest this is not as effective as IPTp [12] [13]. There is debate among researchers and policy-makers on whether we should persist with IPTp using SP in areas where efficacy remains high or switch now to new drugs and/or strategies that may have a long-term future. In areas that already have substantial resistance, replacing SP with dihydroartemisinin-piperaquine is a priority.

## Barriers and challenges in IPTp delivery and implementation

Given that an estimated 75% of pregnant women attend ANC at least twice [14], the low coverage of IPTp and ITNs is concerning and points to broader issues. Low coverage of recommended preventive strategies reflects many challenges to the delivery and implementation of public health interventions in resource-limited settings in sub-Saharan Africa [15]. Key factors include barriers in accessing health services, limited or inconsistent drug supply at health centres, adherence to policy, training and professional development of health care staff, and under-resourced and under-staffed facilities. Community knowledge of IPTp benefits and the cost, convenience, and acceptability of IPTp are also important.

Multiple actions are likely to be needed to improve coverage. This includes strengthening governance, health resourcing and health system strengthening, capacity building, and community engagement [6]. Integration of services is a possible strategy to increase access, but the benefits and potential risks of integration have not been clearly established, and which services can be effectively and practically integrated needs further understanding [16]. Given the major deficiencies in IPTp and ITNs coverage compared to the rates of ANC attendance, implementation of new or additional strategies may be needed. Strengthening commitment from government, national, and regional health departments and national and regional malaria control programs is also important.

## Future/Conclusions

The findings from Walker et al. support prioritising the scale-up of IPTp and ITNs for malaria in pregnancy. However, in light of SP resistance, prioritisation of new drug regimens and monitoring resistance and efficacy are critical. Further operational and implementation research is essential to understand why coverage is so low and to develop and test new strategies and implementation models. Unfortunately, this research tends to be a lower priority for research funding, but it is essential for ensuring that effective interventions widely reach populations at risk. Strengthening the coverage of existing IPTp and ITN interventions will serve as a foundation for the implementation of future interventions. Moreover, commitment to a strong integrated global plan that includes all malaria-endemic regions and prevention of *P*. *falciparum* and *P*. *vivax* malaria is paramount.

## Abbreviations

ANC: antenatal clinic
IPTp: intermittent preventive treatment in pregnancy
ITN: insecticide-treated bed net
LBW: low birth weight
SP: sulphadoxine-pyrimethamine
